# Inhibition of miR-99a-5p prevents allergen-driven airway exacerbations without compromising type-2 memory responses in the intestine following helminth infection

**DOI:** 10.1038/s41385-021-00401-x

**Published:** 2021-04-12

**Authors:** Lewis Entwistle, Helena Aegerter, Stephanie Czieso, Eleni Amaniti, Riccardo Guidi, Abdul Sesay, Nikolay Nikolov, Probir Chakravaty, Alison Huynh, Jessica Mills, Sean Flanagan, Shannon Hambro, Victor Nunez, Yi Cao, Christine Clarke, Angela Martzall, Laurie Leong, Dennis Wilson, Cary Austin, Mark Wilson

**Affiliations:** 1 Francis Crick Institute, London, UK; 2 Immunology, Genentech Inc, South San Francisco, CA USA

## Abstract

Acute exacerbations (AE) of asthma, remain one of the biggest concerns for patients living with asthma. As such, identifying the causes, the molecular mechanisms involved and new therapeutic interventions to prevent AE is a high priority. Immunity to intestinal helminths involves the reactivation of type-2 immune responses leading to smooth muscle contraction and mucus hypersecretion–physiological processes very similar to acute exacerbations in the airways following allergen exposure. In this study, we employed a murine model of intestinal helminth infection, using *Heligmosomoides polygyrus*, to identify miRNAs during active expulsion, as a system for the identification of miRNAs that may contribute to AE in the airways. Concomitant with type-2 immunity and expulsion of *H. polygyrus*, we identified miR-99a-5p, miR-148a-3p and miR-155-5p that were differentially regulated. Systemic inhibition of these miRNAs, alone or in combination, had minimal impact on expulsion of H. *polygyrus*, but inhibition of miR-99a-5p or miR-155-5p significantly reduced house dust mite (HDM)-driven acute inflammation, modelling human acute exacerbations. Immunological, pathological and transcriptional analysis identified that miR-155-5p or miR-99a-5p contribute significantly to HDM-driven AE and that transient inhibition of these miRNAs may provide relief from allergen-driven AE, without compromising anti-helminth immunity in the gut.

## Introduction

Allergic diseases, including rhinitis, eczema and asthma remain a growing global public health concern. Our understanding of disease pathogenesis, and development of therapeutic interventions, has largely focused on targeting type-2 immune responses^1–4^ and in particular at the cytokine, immunoglobulin and cellular level. There remains a need for novel interventions, modalities and targets to treat patients that are not currently met with standard of care (SOC) that do not benefit from the current therapeutics. In particular, a major morbidity associated with asthma is the onset of acute exacerbations (AE), often requiring hospitalisation. As such, the exacerbation frequency, duration, severity and time-to-first exacerbation is frequently used as FDA-approved clinical endpoints for new therapeutic interventions. Although the precise aetiology of AE is broad, including microbial, occupational and environmental irritants, allergen-driven AE are relatively well described, can be modelled accurately in mice and can be translated into clinical settings in allergen challenge studies.^5^

Similar to allergic diseases, helminth infections are highly prevalent worldwide and are in desperate need for novel interventions.^6–9^ Helminths invoke a robust type-2 immune response,^10–12^ which protects the host from patent infections. Studies in helminth-infected rodents and humans has been critical in shaping our understanding of type-2 immunity, including the identification of novel cellular and molecular mechanisms, including **T** helper **(T**_**H**_**)-**1 and T_H_2 cells, type-2 innate lymphoid cells (ILC2)^13,14^ and specialised epithelial cells, such as tuft cells.^15–17^ Many of these observations in helminth-infected mice and patients have subsequently been observed in patients with allergic diseases,^18^ demonstrating the value of studying helminth immunity for both novel anti-helminth interventions, but also to broadly increase our understanding of type-2 immunity that may apply to allergic diseases. In particular, active expulsion of intestinal helminths in animal models involves AE of type-2 immunity,^19^ akin to AE in allergic airways following allergen re-encounter.

Throughout development, differentiation and effector function of both immune and non-immune cells, post-transcriptional regulation of mRNA translation provides a critical phase of cellular regulation. Small non-protein coding RNAs, including miRNAs of approximately 21 nucleotides in length, regulate mRNA either by translational inhibition or mRNA degradation,^20^ providing finite control over gene expression and protein production. As a result, miRNAs have been implicated in a plethora of physiological responses, and are essential in the resolution of many immunological and infectious diseases. We and others have identified miRNAs critical in effector T_H_2 cells,^21,22^ which have a major impact on resulting type-2 immunity, however the role that miRNAs play at regulating type-2 immunity at a broader tissue level is not well described. In addition, miRNAs are emerging as a new class of therapeutics^23^ and represent a novel molecular modality for therapeutic intervention.

In this study, we adopted a well-described model of functional immunity to the natural murine intestinal helminth *Heligmosomoides polygyrus* (also referred to as *Nematospiroides dubius*, *Heligmosomoides polygyrus bakeri* and *Heligmosomoides polygyrus polygyrus*, but here will be referred to as *H. polygyrus*), which induces a robust type-2 immune response,^24^ to characterise the miRNAome in the intestinal tissue. This allowed us to identify candidate miRNAs that are differentially expressed (DE) during these events. We next determined whether these miRNAs were required for type-2 memory responses and anti-helminth immunity and most importantly, determined whether these miRNAs contribute to allergen-driven AE in the airways. Incorporating a variety of experimental controls, we identified miRNAs that were differentially expressed (DE, relative to uninfected control mice) in (i) susceptible mice during acute infection (1°) (Day 7), (ii) resistant mice at early (Day 42, Rx early) and late (Day 63, Rx late) time points and (iii) resistant mice at early (Day 42, 2° early) and late (Day 63, 2° late) time points during active parasite expulsion. Using a series of *in silico* filtering methods and analyses, we focused on 3 miRNAs, miR-155-5p, miR-99a-5p and miR-148a-3p, that were significantly elevated during active expulsion- hypothesising that miRNAs at these time points may have a greater likelihood of contributing to the acute type-2 immune response and/or tissue responses. Therapeutic intervention with systemic miRNA inhibitors, either in combination as a triple-therapy or individually as a mono-therapy, effectively reduced expression of the miRNAs, and despite having little impact on type-2 memory responses in the intestine or parasite expulsion, we significantly reduced house dust mite (HDM)-driven AE in the airways. These data extend the utility of helminth infection models as tools for target discovery, which may be broadly relevant for type-2 immunity in other mucosal sites, such as in the airway, as we show here. Specifically, we identified miR-99a-5p, which was up-regulated in the intestine of helminth resistant mice, remained elevated during active expulsion of *H. polygyrus* and contributed significantly to HDM-driven AE in the airways.

## Results

### Intestinal miRNAs are differentially expressed during acute *H. polygyrus* infection, in mice resistant to secondary *H. polygyrus* infection and during active expulsion

**Fig. 1:**
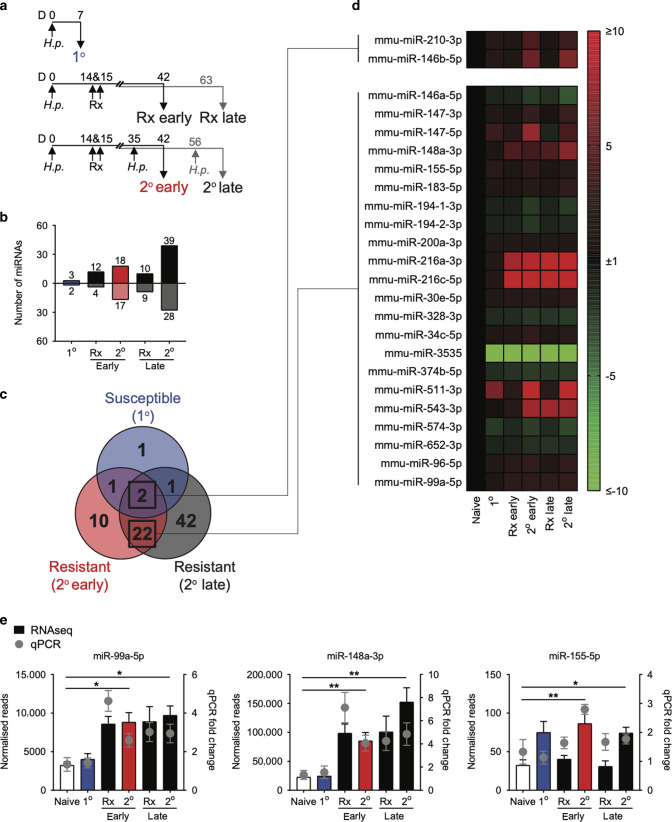
miRNAs are differentially expressed in mice susceptible and resistant to *H. polygyrus*. **a** C57BL/6 mice were orally infected with 200 L3 *H. polygyrus* (*H.p*.) larvae on day 0. A cohort of mice was sacrificed 7 days-post 1° *H. polygyrus* infection (1°). Remaining mice were drug treated (Rx) on days 14 and 15. Two cohorts of mice were culled on days 42 and 63 after drug-cure (Rx early and Rx late, respectively). Two other cohorts of mice were then 2° challenge infected with *H. polygyrus* on day 35 or day 56 and harvested 7 days-post infection (2° early and 2° late, respectively). **b** The number of miRNAs significantly differentially expressed in the small intestine in 1°, Rx early, Rx late, 2° early and 2° late (up- and down-regulated, relative to naïve, *p* < 0.05). **c** Common and differentially expressed miRNAs in susceptible (1°) and resistant (2° early and 2° late) mice (relative to naïve, *p* < 0.05). **d** Expression profile of candidate miRNAs implicated in resistant to *H. polygyrus* identified in (**c**), *n* = 4. **e** miR-99a-5p, miR-148a-3p and miR-155-5p expression in small intestine from RNA sequencing data, *n* = 4, *solid bars*. Significance confirmed by qPCR, *n* = 8, *grey circle*. Data represented as mean ± SEM. *p < 0.05; **p < 0.01, determined using a one-way ANOVA with Dunnett’s multiple comparison analysis.

To characterise the intestinal miRNAome during anti-helminth immunity, we infected C57BL/6 mice with 200 L3 larvae of the natural mouse intestinal helminth *Heligmosomoides polygyrus*.^11^ A cohort of mice were left uninfected, a cohort of mice were analysed at day 7 post 1° infection, designated as susceptible acute infection (1°) (Fig. 1a, top row). The remaining mice were drug-cured (Rx) of the 1° infection on days 14 and 15, which renders mice immune and resistant to a secondary (2°) *H. polygyrus* infection.^24^ A cohort of drug-cured and resistant mice were analysed on day 42 and day 63, designated as resistant early (Rx early, day 42) and resistant late (Rx late, day 63), respectively (Fig. 1a, middle row). Finally, two cohorts of mice were given a 2° challenge infection on day 35 or day 56 and analysed 7 days later, designated as resistant, 2° early and resistant, 2° late, respectively (Fig. 1a, bottom row). Intestinal tissue was analysed 7 days-post 1° or 7 days-post 2° infection, when the larvae are embedded in the intestinal wall (Fig. S1A), as this is the critical period where the worms are trapped and killed in resistant mice^11^ and changes in the intestinal transcriptome associated with susceptibility and resistance are detected.^25^ Resistance to 2° infection was confirmed in a cohort of mice 14 days-post infection where there was a significant reduction in both egg and worm burden (Fig. S1B, C). miRNA sequencing of affected duodenal tissue identified only five significantly DE miRNAs in acute infection (*H.p*. 1°) relative to naïve, uninfected mice (*p* < 0.05). This number increased to 16 and 19 DE miRNAs in resistant mice, following drug-cure, Rx early and Rx late, respectively (relative to naïve, *p* < 0.05). Secondary infection of resistant mice elicited a robust miRNA response with the highest number of DE miRNAs- 35 and 67 miRNAs significantly DE in *H.p*. 2° early and *H.p*. 2° late, respectively (relative to naive, *p* < 0.05) (Fig. 1b). Interestingly, 27-days after drug treatment (Rx early, day 42) we observed 16 DE miRNAs, and 19 DE miRNAs at day 63 (Rx late), 48-days after drug treatment. These data suggest long-term changes in the intestinal miRNAome following intestinal perturbation. To identify miRNAs that may contribute to the robust type-2 response that contributes to resistance, we compared the miRNAome of 1°, 2° early and 2° late (each relative to naive, *p* < 0.05) (Fig. 1c, d). We identified 22 miRNAs that were qualitatively different between susceptible and resistant mice and 2 miRNAs that were common (Fig. 1c, d).

### Elevated expression of three candidate miRNAs, mir-99a-5p, miR-148a-3p and miR-155-5p, correlate with resistance to *H. polygyrus* and are predicted to target 46 DE mRNAs

To confirm the expression and robustness of the 24 candidate miRNAs identified (Fig. 1c, d), we performed qRT-PCR, with increased biological replicates compared to miRNA sequencing (*n* = 8). Candidate miRNAs were identified if their expression was significantly DE (*p* < 0.05) at both 2° early and 2° late, when compared to naive, to increase the likelihood that these miRNAs contributed to resistance. Only three miRNAs passed this more stringent filter: miR-99a-5p, miR-148a-5p and miR-155-5p (Fig. 1e). We, and others, have previously shown that miR-155 is essential for resistance to *H. polygyrus*, with *miR-155*^−/−^ mice failing to expel 2° challenge infection^21^ and that miR-155 is required for allergen-driven airway inflammation.^21,26^ Neither miR-99a-5p or miR-148a-3p have previously been implicated in immunity to intestinal helminth infection or other type-2 immune responses. For all three candidate miRNAs, qRT-PCR validation closely resembled miRNA sequencing expression, confirming two different expression dynamics. Neither miR-99a-5p or miR-148a-3p were significantly DE from naive mice in 1°, but were significantly up-regulated following drug treatment and remained elevated with or without 2° challenge infection (Fig. 1e). In contrast, miR-155-5p expression appeared to be more dynamic and was only significantly DE upon 2° challenge infection, early or late, and then returning to baseline (Fig. 1e). Using RNA-seq data generated from the same samples, and previously reported,^25^ we assessed for putative mRNA targets of miR-99a-5p, miR-148a-5p and miR-155-5p using an in silico (1) predicted miRNA target filter and (2) observed inverse expression data with an expression pairing analysis (relative to naïve, *p* < 0.05) (Fig. S1D). Briefly, DE mRNAs were curated for all time points and used in combination with the miRNA expression profile. For example, predicted mRNA targets of miR-99a-5p (based on target scan and miRBase) that were significantly down-regulated when miR-99a was significantly up-regulated were identified as putative targets. We also required that putative mRNA targets were significantly down-regulated at both 2° early and 2° late time points, when miR-99-5p was significantly up-regulated (Fig. S1E). Of the putative mRNA targets depicted in Fig. S1B, none have previously been associated with anti-helminth immunity. Finally, the differential expression of these miRNAs was confirmed to not be as a result of the drug treatment alone (Fig. S1F). These rich transcriptional data sets, from multiple experimental time points identified three candidate miRNAs, and their predicted mRNA targets, which we hypothesise to contribute to activation of both immune and tissue-mediated expulsion.

### Individual inhibition of miR-99a-5p, miR-148a-3p or miR-155-5p does not impact immunity to *H. polygyrus* infection

**Fig. 2:**
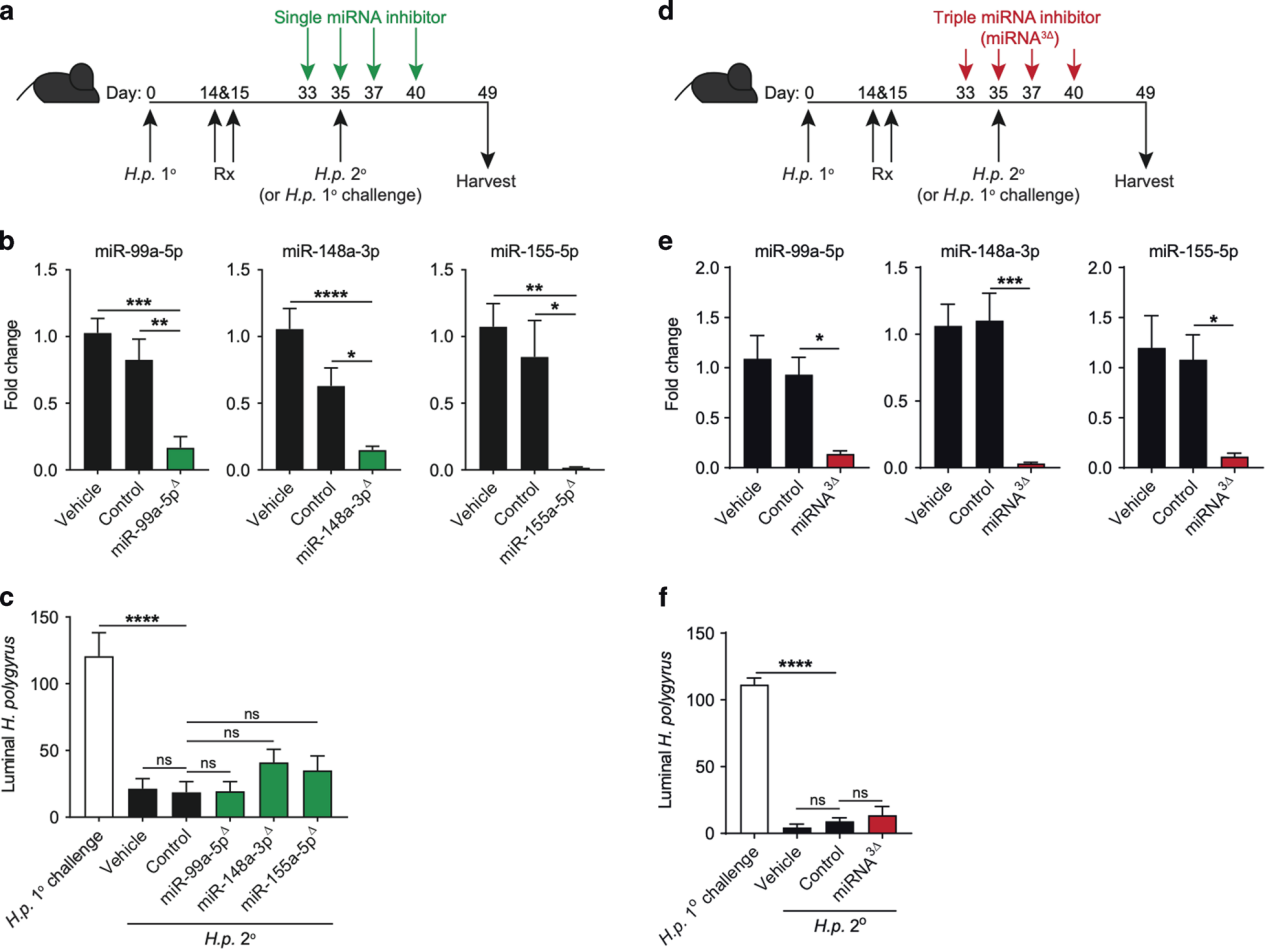
Pharmacological inhibition of miR-99a-5p, miR-148a-3p or miR-155-5p does not perturb immunity to *H. polygyrus*. **a** C57BL/6 mice were orally infected with 200 L3 *H. polygyrus* larvae on day 0 (*H.p*. 1°). Mice were then drug treated (Rx) on days 14 and 15 and 2° challenge infected on day 35 (*H.p*. 2°). Another cohort of C57BL/6 mice were 1° challenge infected only on day 35 (*H.p*. 1° challenge). Mice were treated with miRNA inhibitors, negative control inhibitor or vehicle only on days 33, 35, 37 and 40. Mice were then sacrificed on day 49 (14 days-post 2° infection). **b** miR-99a-5p, miR-148a-5p and miR-155-5p expression following treatment with miRNA-inhibitor (miR-99a-5p^Δ^, miR-148a-5p^Δ^ or miR-155-5p^Δ^), negative control inhibitor (control) or vehicle only. **c** Luminal *H. polygyrus* adult worms in the small intestine 14 days-post 1° challenge or 2° infection following treatment with single miRNA-inhibitor (miR-99a-5p^Δ^, miR-148a-5p^Δ^ or miR-55-5p^Δ^), negative control inhibitor (control) or vehicle only. **d** C57BL/6 mice were orally infected with 200 L3 *H. polygyrus* larvae on day 0 (*H.p*. 1°). Mice were then drug treated (Rx) on days 14 and 15 and 2° challenge infected on day 35 (*H.p*. 2°). Another cohort of C57BL/6 mice were 1° challenge infected only on day 35 (*H.p*. 1° challenge). Mice were treated with triple miRNA inhibitors, negative control inhibitor or vehicle only on days 33, 35, 37 and 40. Mice were then sacrificed on day 49 (14 days-post 2° infection) for analysis. **e** miR-99a-5p, miR-148a-5p and miR-155-5p expression in the small intestine following treatment with the three miRNA inhibitors (miRNA^3Δ^), negative control inhibitor (control) or vehicle only. **f** Luminal *H. polygyrus* adult worms in the small intestine 14 days-post 1° challenge or 2° infection following treatment with the three miRNA inhibitors (miRNA^3Δ^), negative control inhibitor (control) or vehicle only. Data represented as mean ± SEM, *n* = 5 mice per group. All data is representative of two independent experiments. *p < 0.05; **p < 0.01; ***p < 0.001; ****p < 0.0001 determined using a one-way ANOVA with Tukey’s or Dunnett’s multiple comparison analysis.

To test whether any of the candidate miRNA’s, miR-99a-5p, miR-148a-3p or miR-155-5p, contributed to resistance to *H. polygyrus*, we used pharmacological inhibitors of miRNAs 2 days prior to and during 2° challenge infection (Fig. 2a). As all three miRNAs were significantly up-regulated after drug treatment and at least 7 days-post 2° challenge infection (Fig. 1), we treated mice with miRNA inhibitors before (day 33), during (day 35) and after (day 37 and 40) 2° *H. polygyrus* infection to maximise target coverage during the challenge infection period. Treatment with individual miRNA inhibitors, miR-99a-5p^Δ^, miR-148a-3p^Δ^ or miR-155-5p^Δ^ effectively and significantly reduced expression of their respective miRNA targets relative to vehicle treated and scrambled miRNA-inhibitor treatment at day 49 (Fig. 2b). We also confirmed that upon control inhibitor treatment, candidate miRNA expression was not significantly altered, despite a trend to be decreased (Fig. 2b). Control inhibitor treatment had no impact on resistance to 2° challenge *H. polygyrus* infection compared to vehicle treatment (Fig. 2c). Similarly, despite effective inhibition of each candidate miRNA, miR-99a-5p^Δ^, miR-148a-3p^Δ^ and miR-155-5p^Δ^, this had little impact on resistance to 2° challenge infection, with no significant difference seen in luminal worm numbers when compared to control inhibitor treatment (Fig. 2c). Type-2 cytokine production was also completely intact in the mesenteric lymph node (mLN) following inhibitor treatment and 2° challenge infection, with no aberrant cytokine production (Fig. S2A). Furthermore, critical anti-helminth effector molecules, *Arg1, Retnlb* and *Gob5*, and circulating *H. polygyrus*-specific IgG1 titres were unaltered following 2° infection (Fig. S2B–E).

### Triple inhibition of miR-99a-5p, miR-148a-3p and miR-155-5p does not impact immunity to *H. polygyrus* infection

Following the observation that miR-99a-5p, miR-148a-3p and miR-155-5p were all elevated following 2° *H. polygyrus* challenge infection (Fig. 1) and that individual miRNA inhibition had no impact on immunity to *H*. polygyrus (Fig. 2c); we hypothesised that all three miRNAs may act in concert, contributing to anti-helminth effector responses and immunity. To test this hypothesis, we treated mice with all three miRNA inhibitors before (day 33), during (day 35) and after (day 37 and 40) 2° *H. polygyrus* infection (Fig. 2d). Triple miRNA-inhibitor treatment (miRNA^3Δ^) significantly reduced the expression of miR-99a-5p, miR-148a-3p and miR-155-5p in the small intestine on day 49 (14 days-post 2° infection), compared to control inhibitor treatment (Fig. 2e). As demonstrated previously, the control inhibitor-treated mice were resistant to a 2° challenge infection, compared to 1° *H. polygyrus* infection. However, similar to single miRNA inhibition, miRNA^3Δ^ did not abrogate resistance to 2° *H. polygyrus* infection and mice were fully resistant to a 2° challenge infection (Fig. 2f). Again, triple miRNA inhibition did not alter the ability of T cells to produce type 2 cytokines, no aberrant cytokine production was observed (Fig. S3A), and *H. polygyrus*-specific IgG1 responses were similar to control mice (Fig. S3B). These data suggest that despite significantly inhibiting these miRNAs in the target tissue following subcutaneous delivery of miRNA inhibitors from day 33 to day 49, throughout the challenge infection widow, acute inhib**i**tion of these 3 miRNAs (miR-99a-5p, miR-148a-3p and miR-155-5p) does not compromise type-2 immunity in the gut or proficient anti-helminth immunity.

### Inhibition of miR-99a or miR-155-5p abrogates allergic airway inflammation

**Fig. 3:**
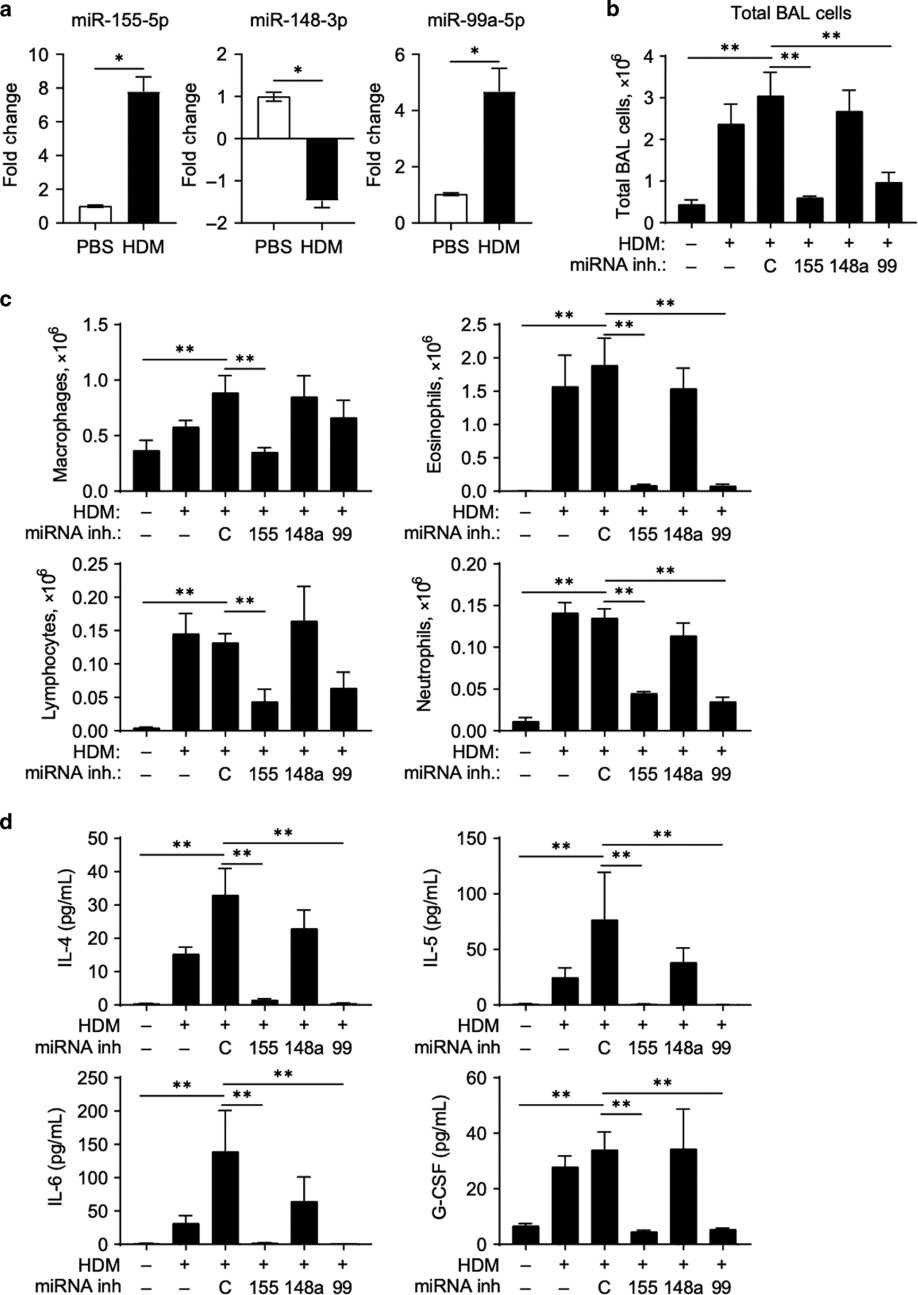
Pharmacological inhibition of miR-99a-5p or miR-155-5p abrogates allergic airway inflammation. **a** miR-99a-5p, miR-148a-5p and miR-155-5p expression in the lung following HDM-induced AE. **b** Total cells recovered from the BAL following treatment with single miRNA-inhibitor (miR-99a-5p, miR-148a-5p or miR-55-5p), negative control inhibitor (**c**) or vehicle only (—) in HDM-induced AAI. **c** Differential cell counts recovered from the BAL. **d** Cytokines detected in the BAL. *n* = *5 mice per group*. All data is representative of two independent experiments. *p < 0.05; **p < 0.01, determined using a Mann–Whitney test.

Our results suggest that upregulation of miRNAs miR-99a-5p, miR-148a-3p and miR-155-5p in mice are not required for resistance to 2° *H. polygyrus* challenge infection in the intestine, despite upregulation during a memory type-2 immune response. To evaluate the role of these miRNAs in the airways following allergen exposure, we modelled an acute allergen-driven exacerbation in mice, by exposing mice to HDM allergen via the intratracheal route on day 0, 2, and 4 to sensitise mice, followed by HDM challenges on day 14 and 16. At this point mice display many hallmarks of allergen-driven airway inflammation. To mimic an allergen-driven acute exacerbation (AE), mice were given a further round of HDM challenges on day 28 and 30 and assessed on day 31 for signs of increased airway inflammation. miR-99a-5p and miR-155-5p, but not miR-148a-3p, were significantly up-regulated in the lung tissue following HDM-induced AE on day 31, compared to PBS-treated controls (Fig. 3a). Administration of control inhibitors prior to allergen re-challenge on days 23 and 25 had no impact on HDM-induced AE with similar cellular infiltration into the BAL (Fig. 3b, c), induction of lung inflammation and goblet cell hyperplasia (Fig. S4A–C), when compared to vehicle control treated mice. However, pharmacological inhibition of miR-155-5p or miR-99a-5p, but not miR-148a-3p, significantly reduced total cell infiltrate into the BAL (Fig. 3b). Specifically, inhibition of miR-155-5p and miR-99a-5p significantly reduced BAL eosinophils and neutrophils, compared to mice treated with control inhibitors (Fig. 3c). BAL cytokines, interleukin (IL)−4, IL-5, IL-6 and G-CSF, were also significantly reduced following inhibition of miR-155-5p and miR-99a-5p (Fig. 3d), suggesting that miR-155-5p and miR-99a-5p contribute to cytokine and chemokine mediated responses, providing a potential explanation for the reduced cellular influx following their inhibition. Inhibition of miR-155-5p or miR-99a-5p also reduced tissue inflammation and goblet cell hyperplasia, as determined by histological scoring (Fig. S4A–C), collectively demonstrating that transient inhibition of miR-155-5p or miR-99a-5p can significantly reduce HDM-driven AE.

### Lung transcriptome analysis reveals profound downregulation of type-2 immunity following miR-99a-5p or miR-155-5p inhibition

**Fig. 4:**
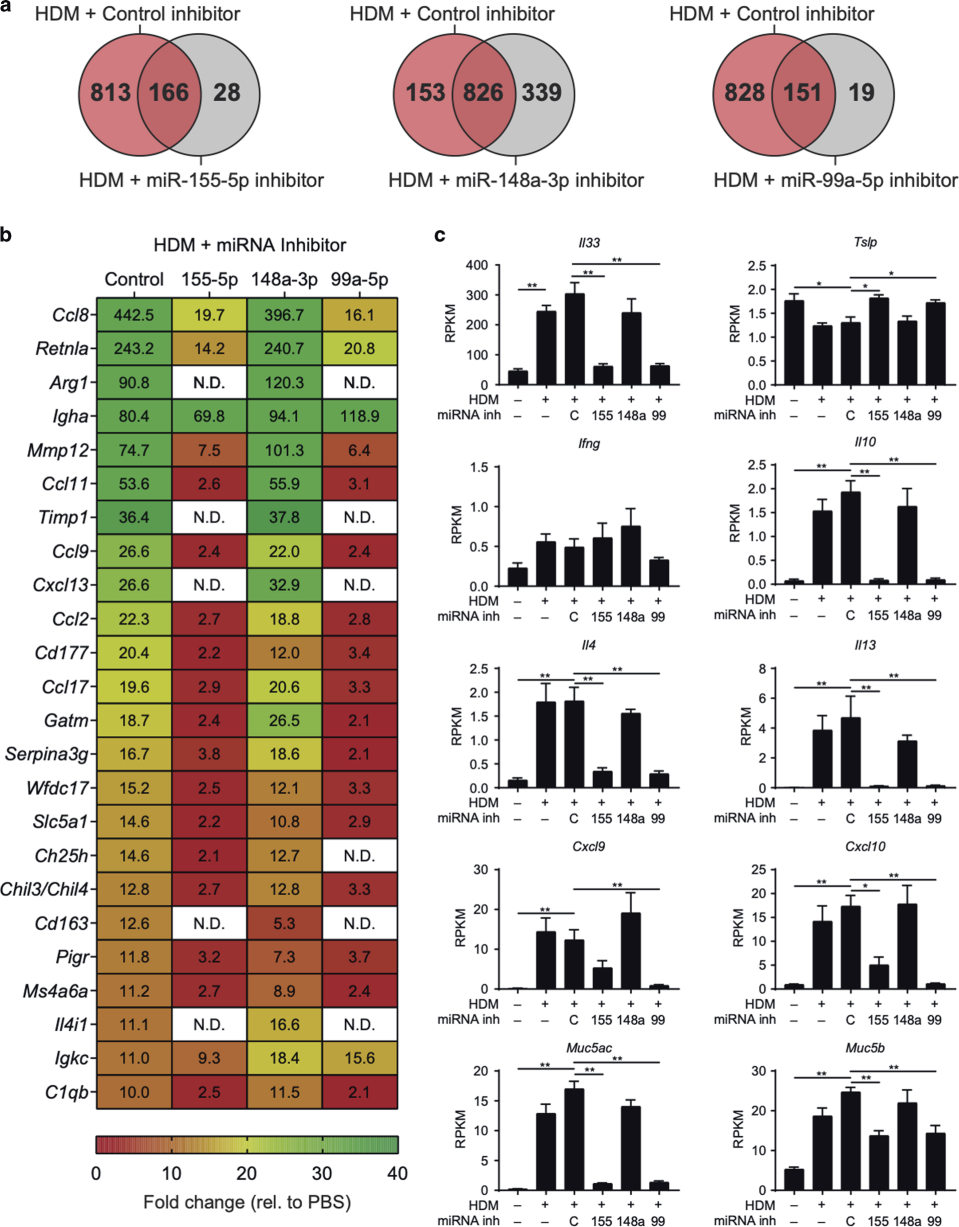
mRNA sequencing reveals miR-99a-5p inhibition alters key type-2 immune pathways in the lung during AAI. **a** The number of common and distinct differentially expressed genes in the lungs of HDM-treated mice that received individual miRNA-inhibitor treatment (relative to PBS control, RPKM > 1, FDR > 0.05). **b** The top 24 most up-regulated differentially expressed genes in the lungs of control inhibitor-treated HDM mice and their expression in miRNA-inhibitor-treated HDM mice (Fold change relative to PBS, RPKM > 1, FDR > 0.05), N.D. no difference. **c** Selected expression profiles of key genes in allergic airway inflammation following HDM challenge and miRNA-inhibitor treatment. *n* = 5 mice per group from one experiment. *p < 0.05; **p < 0.01, determined using a Mann–Whitney test. N.D. denotes not differentially expressed.

To identify putative mechanisms following inhibition of miR-155-5p or miR-99a and to obtain some insights of why miR-148a-3p inhibition did not impact HDM-driven AE, we assessed the transcriptional landscape in the lung of HDM-treated mice that had been treated with or without miRNA inhibitors. Pharmacological inhibition of miR-155-5p or miR-99a-5p dramatically reduced the number of DEG (196 and 170 DEG, respectively, relative to PBS control) compared to control inhibitor treatment (979 DEG) (Fig. 4a), whereas miR-148a-3p inhibition largely overlapped with control inhibitor treatment, with 1165 DEG (Fig. 4a, middle Venn diagram). miR-155-5p inhibitors or miR-99a-5p inhibitors significantly inhibited many of the most up-regulated genes in HDM exposed mice, whereas miR-148a-3p inhibitors had little impact (Fig. 4b). These included key type-2 effector genes such as *Retnla* (Relm-α), *Arg1* (Arginase-1) and *Ccl11* (Eotaxin-1). The putative miR-99a mRNA targets identified in the small intestine (Fig. S1) were largely unaffected in the lung following HDM challenge with or without miRNA inhibition (data not shown), suggesting that organ specific responses are elicited and that miR-99a may have different mRNA targets in different tissues. Analysis of key mediators of type-2 immunity identified that expression of *Il33*, a critical alarmin cytokine in the lung, was also significantly inhibited in miR-155-5p and miR-99a-5p inhibitor-treated mice, compared to control inhibitor-treated mice (Fig. 4c), suggesting a failure to properly initiate type-2 immunity. The expression of *Il10*, *Il4* and *Il13* as well as the mucin genes *Muc5ac* and *Muc5b*, were also significantly reduced in miR-155-5p and miR-99a-5p treated mice, suggesting that type-2 effector responses failed to develop, in line with reduced protein secretion in the BAL and following *ex* vivo re-stimulation (Figs. 3, S4D) and reduced goblet cell hyperplasia (Fig. S4C). Of note, *Ifng* expression was largely unaltered in the lung tissue following miRNA inhibition (Fig. 4c). However IFN*-*regulated chemokines *Cxcl9* and *Cxcl10*, both of which have been observed in the airways of patients with severe asthma,^27,28^ were reduced following miR-155-5p and miR-99a-5p inhibition.

Pathway analysis identified that both innate, adaptive and a variety of signalling pathways are reduced following miR-155-5p and miR-99a-5p inhibition (Fig. S5). Taken together, this immunological, pathological and transcriptional analysis suggests that miR-155-5p and miR-99a-5p contribute significantly to HDM-driven AE and that transient inhibition of these miRNAs may provide relief from allergen-driven exacerbations of airway disease, without compromising type-2 memory responses in the intestine, which are required for anti-helminth immunity.

## Discussion

Reactivation of memory T_H_2 cells and episodic phases of type-2 immunity are associated with a wide range of diseases and infections across a variety of different tissues. Studies in helminth-infected rodents and humans have been critical for understanding type-2 immunity and identified novel mechanisms that are relevant for patients with allergic diseases. This demonstrates the value of studying helminth immunity for both novel anti-helminth interventions and increasing our understanding of type-2 immunity that has applications in allergic diseases.

In this study, we provide data that underlines the utility of helminth infection models as a tool for novel target discovery in type-2 immune-mediated mucosal disorders. Specifically, using an established mouse model of anti-helminth immunity, we identified that miR-99a-5p and miR-155-5p were up-regulated in the intestine of helminth-infected mice and remained elevated during active expulsion of *H. polygyrus*. Inhibition of either, or both, miR-99a-5p and miR-155-5p had no significant impact on memory type-2 immune responses in the intestine and did not impact anti-helminth immunity. However, we confirmed a role for miR-155, and identified an essential role for miR-99a-5p in HDM-driven airway inflammation. These data suggest that transient pharmacological inhibition of miR-99a-5p, or miR-155, may prevent allergen-driven exacerbations of airway disease, without compromising memory type-2 immune responses in the intestine or anti-helminth immunity.

We, and others, have previously identified elevated expression of miR-155 in the intestine of helminth-infected mice, in the lung of allergen-challenged mice and specifically established a critical role for mir-155 in T_H_2 cells contributing to these responses.^21,26^ We therefore anticipated that miR-155 would be elevated in this study, in both the gut (Fig. 1f) and lung (Fig. 3a) and that it would pass our filtering criteria as a ‘candidate’ miRNA. However, it was unexpected that despite significant inhibition of miR-155-5p (98% inhibition) in the intestine throughout the secondary challenge period (day 33 to day 49) following subcutaneous delivery of the inhibitors, that we did not abrogate protective type-2 immunity in the gut.

Studies, including our own,^21^ using *miR155*^−/−^ mice and mice with *miR155*^−^^/−^ T cells, would have predicted that transient inhibition of miR-155 would compromise anti-helminth immunity. Both spatial and temporal differences between transient pharmacological inhibition and genetic deletion may explain these differences and highlight a point of caution when attempting to translate an observation made in gene-deficient animals to therapeutic intervention.^29^ Specifically, in this case, miR-155 has broad functions throughout T-cell development and effector function in a variety of settings^30–35^ and reviewed here,^36^ that genetic deletion of miR-155 may have such profound impacts rendering T cells broadly compromised. In our study, we observed ~85–95% inhibition in tissue biopsies (Fig. 2), without any impact on T-cell recall responses from local lymph nodes (Fig. S2), suggesting that either we did not achieve sufficient miR-155 inhibition in T cells, and other critical immune and non-immune cells, or that once both tissue^25^ and memory immune cells have developed, miR-155 is no longer required. Extending these speculations to miR-99a and miR-148a, it would be of great interest to generate miR-99a and miR-148a-deficient mice to assess whether genetic deletion of these miRNAs would abrogate immunity to *H. polygyrus*, similar to *miR155*^–/–^ mice. It is possible that alternative routes of administration, including localised delivery, may increase the biodistribution of the miRNA inhibitors in the intestinal tissue and achieve greater than 98% miRNA inhibition or prolong target coverage. An alternative explanation of why the individual miRNA inhibition did not abrogate type-2 memory responses in the intestine or protective anti-helminth immunity was that there was redundancy between miR-99a-5p, miR-148a-3p and miR-155-5p and that perturbation of only a single miRNA would not be sufficient to abrogate resistance. However, concurrent inhibition of all three miRNAs did not abrogate protective anti-helminth immunity.

Despite having no effect on anti-helminth immunity, we identified for the first time that miR-99a-5p was required for allergen-driven airway inflammation and that pharmacological inhibition of miR-99a-5p or miR-155-5p was successful in abrogating allergen-driven type-2-associated inflammation and pathology. Similar to helminth-infected mice, inhibition of miR-99a-5p and miR-155-5p was administered therapeutically, following several rounds of HDM-driven airway inflammation. These data are in line with and extend our previous studies on miR-155 in T_H_2 cells in airway inflammation.^21^ We cannot conclude that the mechanism of action of these pharmacological inhibitors is mediated by T cells alone, especially as miR-155 has broad roles in a variety of cells, including macrophages, DCs and B cells.^37–40^ Nevertheless, these data raise the possibility that transient pharmacological inhibition of miR-155 may be a viable therapeutic option in allergen-induced asthma that merits further study.

miR-99a has not been studied in the context of type-2 immunity, but has been reported to regulate inflammatory cytokine production, via targeting mTOR.^41–44^ Studies in human endothelial cell lines demonstrated that NF-κB-induced miR-99a regulates LPS-induced inflammation, with miR-99a overexpression inhibiting inflammatory cytokine production in a negative feedback loop, likely through mTOR suppression.^42^ MiR-99a overexpression in γδ T cells inhibited γδ T-cell activation and promoted apoptosis.^44^ Similarly, miR-99a has been observed in CD4^+^ T cells, with overexpression of miR-99a in T cells in vitro supporting regulatory T-cell (T_REG_) differentiation and slightly inhibiting T_H_17 differentiation, potentially via repression of mTOR.^41^ Treatment of HDM-challenged mice with miR-99a inhibitors did not result in any measurable IL-17A in the BAL or re-stimulated lymph nodes (data not shown), suggesting that miR-99a inhibition did not dramatically shift the T-cell response in our studies. Previous studies suggest that miR-99a can suppress Type 1- or LPS-induced inflammation.^42,43^ Following this line of research, one possible mechanism in our model is that inhibition of miR-99a could allow an inappropriate Type-1 or Type 17 immune response to develop, preventing HDM-driven Type-2 immunity. However, our immunological and transcriptomic analyses do not support this; instead, transcriptional analysis of lung tissue implicated a broad dampening of cytokine production mirrored by a general inhibition of innate and adaptive responses, along with a variety of signalling pathways (Fig. S5), rather than an aberrant Type 1 or Type 17 response. miR-99a-5p has also been observed in neutrophils,^45^ but its function in neutrophils is unclear. Whether perturbing neutrophil function via inhibition of miR-99-5p, in this setting of HDM-driven AE would have such profound consequences on downstream type-2 immunity is unlikely, but as yet untested.

More likely, miR-99a inhibition may have inhibited upstream innate immune cell drivers of reactivation and type-2 immunity, including airway macrophages^46,47^ and epithelial cells^48^ where miR-99a inhibition had an impact. Elevated miR-99a expression has previously been observed in IL-4 treated ‘M2’ macrophages when compared to untreated ‘M0’ or LPS/IFNγ-treated ‘M1’ macrophages.^43^ Furthermore, miR-99a appears to be required for M2 macrophage activation, with miR-99a knockdown experiments abolishing an M2 phenotype and with reduced expression of Relm-α, Arginase-1 and Ym-1, and miR-99a overexpression studies preventing M1 activation, including *Inos*, *Mcp1* and *Il1b* expression. Indeed, we observed a dramatic reduction in M2 macrophage markers, *Retnla* (*Fizz1*), *Arg1* and *Chil3* (Ym1) in miR-99a inhibitor-treated mice (Fig. 4b). Mechanistically, expression of critical chemokines produced from macrophages, such as *Ccl8* and *Ccl9*, were significantly reduced in the lung tissue following miR-99a-5p inhibition. Ccl8 is a potent chemoattractant for pathogenic T_H_2 cells and ILC2s,^49,50^ whereas Ccl9 is capable of recruiting monocytes, neutrophils and eosinophils via Ccr1.^51^

miR-99a-5p inhibitor treatment also down-regulated the expression of *Il33*, but had no impact on production of IL-25 or thymic stromal lymphopoietin (TSLP). Inhibition of *Il33* may prevent downstream activation of ILC2 and lymphocytes,^52^ preventing HDM-driven AE. This may also provide an explanation as to why miR-99a-5p inhibition was successful in preventing type-2 immunity in the lung, but preserved type-2 responses in the gut; alarmins have been demonstrated to have more dominant roles in activating resident ILC2s depending on the tissue, with IL-33 signalling enriched in the lung and IL-25 in the gut.^53^

In conclusion, using the expulsion of *H. polygyrus* to model acute reactivation of type-2 immune responses, we identified a series of miRNAs, including miR-99a-5p, miR-155-5p and miR-148a-3p that were up-regulated during active expulsion. miR-99a-5p, and miR-155-5p were also up-regulated in the lung of mice following HDM-driven acute exacerbation. Pharmacological inhibition of these miRNAs, alone or in combination, did not compromise anti-helminth immunity, but inhibition of miR-99a-5p or miR-155-5p significantly prevented HDM-driven AE in the lung, as assessed at the immunological, pathological and transcriptional level. Targeting these miRNAs may have advantages over targeting cytokines, cytokine receptors, or defined signalling pathways, by virtue of the multiple pathways that are regulated by miRNAs. Understanding the critical cellular targets, tissue expression and precise mechanism of action of these miRNA inhibitors could support the clinical development of these novel therapies for type-2, allergen-driven AE of asthma.

## Materials and methods

### Animal strains

All mice (C57BL/6J) used in this study were maintained under specific pathogen-free conditions at either The Francis Crick Institute (London, UK) or at Genentech (South San Francisco, USA). C57BL/6J mice used at The Francis Crick Institute were bred in house. C57BL/6J mice used at Genentech were purchased from Jackson Laboratories. Mice were kept in a standard 12 h light–dark cycle and were allowed free access to sterile food and water. Experiments were performed according to institutional guidelines following UK Home Office regulations (project license 70/8809) and were approved by The Francis Crick Institute Ethical Review Panel or were approved by the Laboratory Animal Resources Committee (LARC) at Genentech, Inc. and adhered to the NIH Guidelines for the Care and Use of Laboratory Animals.

### Helminth infection model and miRNA inhibition

Mice were infected with 200 L3 infective *H. polygyrus* larvae *(p.o*.) on day 0 (1° infection). Mice were drug-cured (Rx) with the anthelminthic drug Pyrantel Embonate (2.5 mg/dose, Pfizer) *(p.o*.) on days 14 and 15. Mice were secondary (2°) challenge infected on day 35 or day 56 with 200 L3 infective *H. polygyrus* larvae *(p.o*.). Luminal *H*. polygyrus worms were counted 14 days-post 1° or 2° infection. In vivo miRCURY LNA^TM^ microRNA Inhibitors (Exiqon) were designed and manufactured for inhibiting mmu-miR-99a-5p, mmu-miR-148a-3p and mmu-miR-155-5p. A negative control in vivo miRCURY LNA^TM^ microRNA inhibitor (Exiqon) was also used. miRNA inhibitors or a PBS vehicle control were administered prior to and during 2° challenge infection on days 33, 35, 37 and 40 (0.125 mg per dose, *s.c*.).

### Acute allergic airway inflammation exacerbation model and miRNA inhibition

Mice were sensitised with HDM (10 µg, Greer) via passive intratracheal inhalation, (p.i.t.i.) on day 0, 2 and 4. Sensitised mice were challenged with PBS or HDM (10 µg, Greer) on days 14 and 16 to establish airway inflammation. Mice were then re-challenged with PBS or HDM (10 µg, Greer) on 28 and 30 to induce an allergen-driven acute exacerbation. Mice were culled for analysis 24 h following the final re-challenge. In vivo miRCURY LNA^TM^ microRNA Inhibitors (Exiqon) or a PBS vehicle control were administered prior to allergen re-challenge on days 23 and day 25 (0.125 mg per dose, *s.c*.). Total BAL cells were collected in 2 BAL washes. Firstly, with 500 µl of sterile cold PBS for analyte analysis and cellular recovery and then with 1 ml of sterile cold PBS for cellular recovery.

### Histology

Small intestinal tissue and lung tissue was removed and fixed in 4% formaldehyde for 24 h then washed in 70% ethanol. The tissues were embedded in paraffin, and sectioned. Sections were stained with hematoxylin and eosin stain or Alcian blue/periodic acid-Schiff stain for analysis.

### RNA extraction

Tissues were harvested and stored in RNAlater (Sigma) for 24 h at 4 °C before storage at −80 °C. For RNA extraction, RNAlater was removed and tissue was homogenised in Qiazol (Qiagen) using the Precellys homogeniser (Bertin Instruments). Cells were lysed and stored in Qiazol (Qiagen) at −80 °C. RNA was extracted using the miRNeasy Kit (Qiagen), following the manufacturer’s instructions. RNA concentration was measured using a ND-1000 Spectrophotometer (NanoDrop Technologies) or Qubit 2.0 Fluorometer (Invitrogen).

### Quantitative real-time polymerase chain reaction

For mRNA analysis, reverse transcription was performed with 0.1–1 µg RNA using Qiagen^®^ Quantitect RT Kit following manufacturer’s instructions to create cDNA. For miRNA analysis, reverse transcription was performed with 0.1–1 µg RNA using miSCRIPT II RT Kit, HiSpec buffer, (Qiagen) following manufacturer’s instructions to create cDNA. Generated cDNA was used for quantitative real-time PCR analysis using Power SYBR^®^ Green PCR Master Mix (Applied Biosystems) and quantified on the 7900HT (Applied Biosystems) or QuantStudio5 (Applied Biosystems). See Table S1 for mRNA primer sequences. Where appropriate, relative mRNA expression was determined via normalisation to the housekeeping gene *Hprt* and the relevant control group (see Figure legends). All miRNA and snoRNA primers were purchased from Qiagen for use with the miSCRIPT II RT Kit. Where appropriate, relative miRNA expression was determined via normalisation to the housekeeping snoRNA RNU6B and the relevant control group (see Figure legends).

### RNA sequencing and analysis

#### miRNAseq

RNA integrity was confirmed using Agilent’s 2100 Bioanalyser. miRNA libraries were created using the NEBNext Multiplex Small RNA Library Prep Set for Illumina (New England BioLabs), following manufacturer’s instructions. miRNA libraries were sequenced using the Illumina^®^ MiSeq. The raw Illumina reads were analysed as follows. First, the data quality was analysed using FastQC (www.bioinformatics.babraham.ac.uk/projects/fastqc). Low quality bases were trimmed using Trimmomatic. The read pairs which passed the trimming quality filters were then aligned to mirBase (release21) using Novoalign v3.02.12 (http://www.novocraft.com/support/download/). Normalisation and statistical analysis was performed using edgeR script. Differential gene analysis was calculated from naïve control group. Statistically significant miRNAs with FDR < 0.05 were reported. *RNA sequencing and gene expression profiling:* Whole-transcriptome profiles were generated using TruSeq RNA Access technology (Illumina). RNA-seq reads were processed using the HTSeqGenie R package (v. 4.2.2). Briefly, RNA-seq reads were first aligned to ribosomal RNA sequences to remove ribosomal reads. The remaining reads were aligned to the mouse reference genome (GRCm38) using GSNAP (1,2) version 2013-11-10, allowing a maximum of two mismatches per 75 base sequence (parameters: ‘-M 2 -n 10 -B 2 -i 1 -N 1 -w 200,000 -E 1-pairmax-rna = 200,000 –clip-overlap). To quantify gene expression levels, we counted the number of reads aligning within exons of gene models provided by GENCODE basic (v. 27).

### Cell isolation

Spleen and mLNs were made into single-cell suspensions by gently mashing through a 40 mM filter (Thermo-Scientific, Loughborough, UK), and the red blood cells were lysed from the spleen single-cell suspension with ACK lysis buffer (Gibco). Single cell suspensions were used for ex vivo restimulations and flow cytometry analysis.

### Flow cytometry and cell sorting

Cell suspensions were stained for 25 min with antibodies in PBS with 1% FCS. For flow cytometry analysis, cells were analysed using a BD LSRFortessa™ X-20 (BD Biosciences) or BD LSRII (BD Biosciences) and data were analysed using FlowJo software (Version 10, Treestar Inc). Cells were sometimes fixed in 2–4% paraformaldehyde for FACS analysis. Viability of the cells was determined using the LIVE/DEAD Fixable Blue kit (Life Technologies). Antibodies used include: CD4 (RM4-5; efluor450 (eBioscience)), CD44 (IM7; Percpcy5.5 (eBioscience)), IFNγ (XMG1.2; PE (BD Bioscience)), IL4 (PE, 11B11, eBioscience)), IL5 (APC, TRFK5, BD Bioscience)), IL13 (eFluor660, eBio13A, eBioscience)), IL17a (17B7; PE-Cy7 (eBioscience)), All staining was performed in the presence of FcR Blocking Reagent (Miltenyi Biotec). Intracellular cytokine staining (ICS) was performed following 6 h of re-stimulation with 50 ng/mL phorbol 12-myristate 13-acetate (PMA, Promega) and 1 µg/mL ionomycin (Sigma) and BD Golgi Stop and BD Golgi Plug (diluted 1:1000, BD Biosciences). Following surface stain, cells were incubated with eBioscience Fixation/Permeabilization buffer for 25 min followed by 25 min in Permeabilization buffer (eBioscience), and incubation with antibodies in Permeabilization buffer for a further 30 min.

### Ex vivo stimulations

mLNs were harvested and processed as above. Cells were plated at 2 × 10^5^ cells per 200 µl and stimulated with 10 µg HDM (Greer) for 3 days Supernatant was removed and secreted cytokines were analysed.

### ELISAs and Luminex

*H*. *polygyrus-*specific IgG1 was detected by coating plates with 5 μg/mL *H*. *polygyrus* antigen overnight, followed by overnight incubation with serially diluted serum and detection with Biotin Rat Anti-Mouse IgG1 (Invitrogen) and Streptavidin HRP at 1:000 (BD Pharmingen) and ABTS One Component HRP Microwell Substrate (SurModics). Cytokines in BAL were analysed by Luminex.

### Statistical analysis

All statistical analysis for biological data was performed using GraphPad Prism (v8). Data was analysed with either, where appropriate; unpaired two-tailed *t* test, One-way ANOVA (Dunnett’s multiple comparison analysis), Two-way ANOVA (Sidak’s multiple comparison analysis) or Mann–Whitney test. Values are reported as the means ± SEM. *p < 0.05; **p < 0.01; ***p < 0.001; ****p < 0.0001.

## Supplementary information

Supplementary information

## References

[1] Corren J (2017). Tezepelumab in adults with uncontrolled asthma. N. Engl. J. Med..

[2] Haldar P (2009). Mepolizumab and exacerbations of refractory eosinophilic asthma. N. Engl. J. Med.

[3] Rabe KF (2018). Efficacy and safety of dupilumab in glucocorticoid-dependent severe asthma. N. Engl. J. Med..

[4] Schulman ES (2001). Development of a monoclonal anti-immunoglobulin E antibody (omalizumab) for the treatment of allergic respiratory disorders. Am. J. Respir. Crit. Care Med..

[5] Singh AM, Busse WW (2006). Asthma exacerbations. 2: aetiology. Thorax.

[6] Hotez PJ (2008). Helminth infections: the great neglected tropical diseases. J. Clin. Investig..

[7] Bethony J (2006). Soil-transmitted helminth infections: ascariasis, trichuriasis, and hookworm. Lancet.

[8] Albonico M (2003). Efficacy of mebendazole and levamisole alone or in combination against intestinal nematode infections after repeated targeted mebendazole treatment in Zanzibar. Bull. World Health Organ..

[9] Taman A, Azab M (2014). Present-day anthelmintics and perspectives on future new targets. Parasitol. Res..

[10] Allen JE, Maizels RM (2011). Diversity and dialogue in immunity to helminths. Nat. Rev. Immunol..

[11] Reynolds LA, Filbey KJ, Maizels RM (2012). Immunity to the model intestinal helminth parasite Heligmosomoides polygyrus. Semin. Immunopathol..

[12] Maizels RM, Hewitson JP, Smith KA (2012). Susceptibility and immunity to helminth parasites. Curr. Opin. Immunol..

[13] Neill DR (2010). Nuocytes represent a new innate effector leukocyte that mediates type-2 immunity. Nature.

[14] Fallon PG (2006). Identification of an interleukin (IL)−25-dependent cell population that provides IL-4, IL-5, and IL-13 at the onset of helminth expulsion. J. Exp. Med..

[15] Gerbe F (2016). Intestinal epithelial tuft cells initiate type 2 mucosal immunity to helminth parasites. Nature.

[16] Howitt MR (2016). Tuft cells, taste-chemosensory cells, orchestrate parasite type 2 immunity in the gut. Science.

[17] von Moltke J (2016). Tuft-cell-derived IL-25 regulates an intestinal ILC2-epithelial response circuit. Nature.

[18] Smith SG (2016). Increased numbers of activated group 2 innate lymphoid cells in the airways of patients with severe asthma and persistent airway eosinophilia. J. Allergy Clin. Immunol..

[19] Anthony RM (2007). Protective immune mechanisms in helminth infection. Nat. Rev. Immunol..

[20] Ambros V (2003). A uniform system for microRNA annotation. RNA.

[21] Okoye IS (2014). Transcriptomics identified a critical role for Th2 cell-intrinsic miR-155 in mediating allergy and antihelminth immunity. Proc. Natl Acad. Sci. U.S.A..

[22] Pua HH (2016). MicroRNAs 24 and 27 suppress allergic inflammation and target a network of regulators of T helper 2 cell-associated cytokine production. Immunity.

[23] Chakraborty C (2017). Therapeutic miRNA and siRNA: moving from bench to clinic as next generation medicine. Mol. Ther. Nucleic Acids.

[24] Finkelman FD (1997). Cytokine regulation of host defense against parasitic GI nematodes: Lessons from studies with rodent models. Annu. Rev. Immunol..

[25] Entwistle LJ (2017). Epithelial-cell-derived phospholipase A2 Group 1B is an endogenous anthelmintic. Cell Host Microbe.

[26] Malmhall C (2014). MicroRNA-155 is essential for T(H)2-mediated allergen-induced eosinophilic inflammation in the lung. J. Allergy Clin. Immunol..

[27] Hartl D (2005). Pulmonary chemokines and their receptors differentiate children with asthma and chronic cough. J. Allergy Clin. Immunol..

[28] Miotto D (2001). Expression of IFN-gamma-inducible protein; monocyte chemotactic proteins 1, 3, and 4; and eotaxin in TH1- and TH2-mediated lung diseases. J. Allergy Clin. Immunol..

[29] Guidi, R., Wedeles, C. J. & Wilson, M. S. ncRNAs in Type-2 Immunity. *Noncoding RNA* **6**, 10 (2020). 10.3390/ncrna6010010 PMC7151598 32155783

[30] Escobar T (2013). STAT3 activates miR-155 in Th17 cells and acts in concert to promote experimental autoimmune uveitis. Investig. Ophthalmol. Vis. Sci..

[31] Lind EF, Elford AR, Ohashi PS (2013). Micro-RNA 155 is required for optimal CD8+ T cell responses to acute viral and intracellular bacterial challenges. J. Immunol..

[32] Oertli M (2011). MicroRNA-155 is essential for the T cell-mediated control of Helicobacter pylori infection and for the induction of chronic gastritis and colitis. J. Immunol..

[33] O’Connell RM (2010). MicroRNA-155 promotes autoimmune inflammation by enhancing inflammatory T cell development. Immunity.

[34] Almanza G (2010). Selected microRNAs define cell fate determination of murine central memory CD8 T cells. PLoS ONE.

[35] Kohlhaas S (2009). Cutting edge: the Foxp3 target miR-155 contributes to the development of regulatory T cells. J. Immunol..

[36] Lind EF, Ohashi PS (2014). Mir-155, a central modulator of T-cell responses. Eur. J. Immunol..

[37] O’Connell RM (2007). MicroRNA-155 is induced during the macrophage inflammatory response. Proc. Natl Acad. Sci. U.S.A..

[38] Vigorito E (2007). microRNA-155 regulates the generation of immunoglobulin class-switched plasma cells. Immunity.

[39] Teng G (2008). MicroRNA-155 is a negative regulator of activation-induced cytidine deaminase. Immunity.

[40] Zech A (2015). MicroRNA-155 modulates P2R signaling and Th2 priming of dendritic cells during allergic airway inflammation in mice. Allergy.

[41] Warth SC (2015). Induced miR-99a expression represses Mtor cooperatively with miR-150 to promote regulatory T-cell differentiation. EMBO J..

[42] Bao MH (2016). NF-kappaB-regulated miR-99a modulates endothelial cell inflammation. Mediators Inflamm..

[43] Jaiswal A (2019). MicroRNA-99a mimics inhibit M1 macrophage phenotype and adipose tissue inflammation by targeting TNFalpha. Cell Mol. Immunol..

[44] Zhu Y (2019). miR-125b-5p and miR-99a-5p downregulate human gammadelta T-cell activation and cytotoxicity. Cell Mol. Immunol..

[45] Zhao H (2017). MicroRNA-99a-5p in circulating immune cells as a potential biomarker for the early diagnosis of ischemic stroke. Brain Circ..

[46] Mathie SA (2015). Alveolar macrophages are sentinels of murine pulmonary homeostasis following inhaled antigen challenge. Allergy.

[47] Yamaguchi M (2018). Macrophages regulate lung ILC2 activation via Pla2g5-dependent mechanisms. Mucosal Immunol..

[48] Lloyd CM, Saglani S (2010). Asthma and allergy: the emerging epithelium. Nat. Med..

[49] Islam SA, Luster AD (2012). T cell homing to epithelial barriers in allergic disease. Nat. Med.

[50] Puttur, F. et al. Pulmonary environmental cues drive group 2 innate lymphoid cell dynamics in mice and humans. *Sci. Immunol*. **4**, eaav7638 (2019). 10.1126/sciimmunol.aav7638 PMC6744282 31175176

[51] Maurer M, von Stebut E (2004). Macrophage inflammatory protein-1. Int J. Biochem. Cell Biol..

[52] Schmitz J (2005). IL-33, an interleukin-1-like cytokine that signals via the IL-1 receptor-related protein ST2 and induces T helper type 2-associated cytokines. Immunity.

[53] Ricardo-Gonzalez, R. R. et al. Tissue signals imprint ILC2 identity with anticipatory function. *Nat. Immunol.* **19**, 1093–1099 (2018). 10.1038/s41590-018-0201-4 PMC6202223 30201992

