# Supplementary material for: Inhibition of miR-99a-5p prevents allergen-driven airway exacerbations without compromising type-2 memory responses in the intestine following helminth infection
Source: Mucosal Immunol. 2021 Apr 12;14(4):912–22. doi: 10.1038/s41385-021-00401-x (PMC8222002; doi:10.1038/s41385-021-00401-x)
Supplement: Supplementary file 1 — Supplementary information [file 41385_2021_401_MOESM1_ESM.pdf]

Figure S1: Candidate miRNA putative mRNA targets

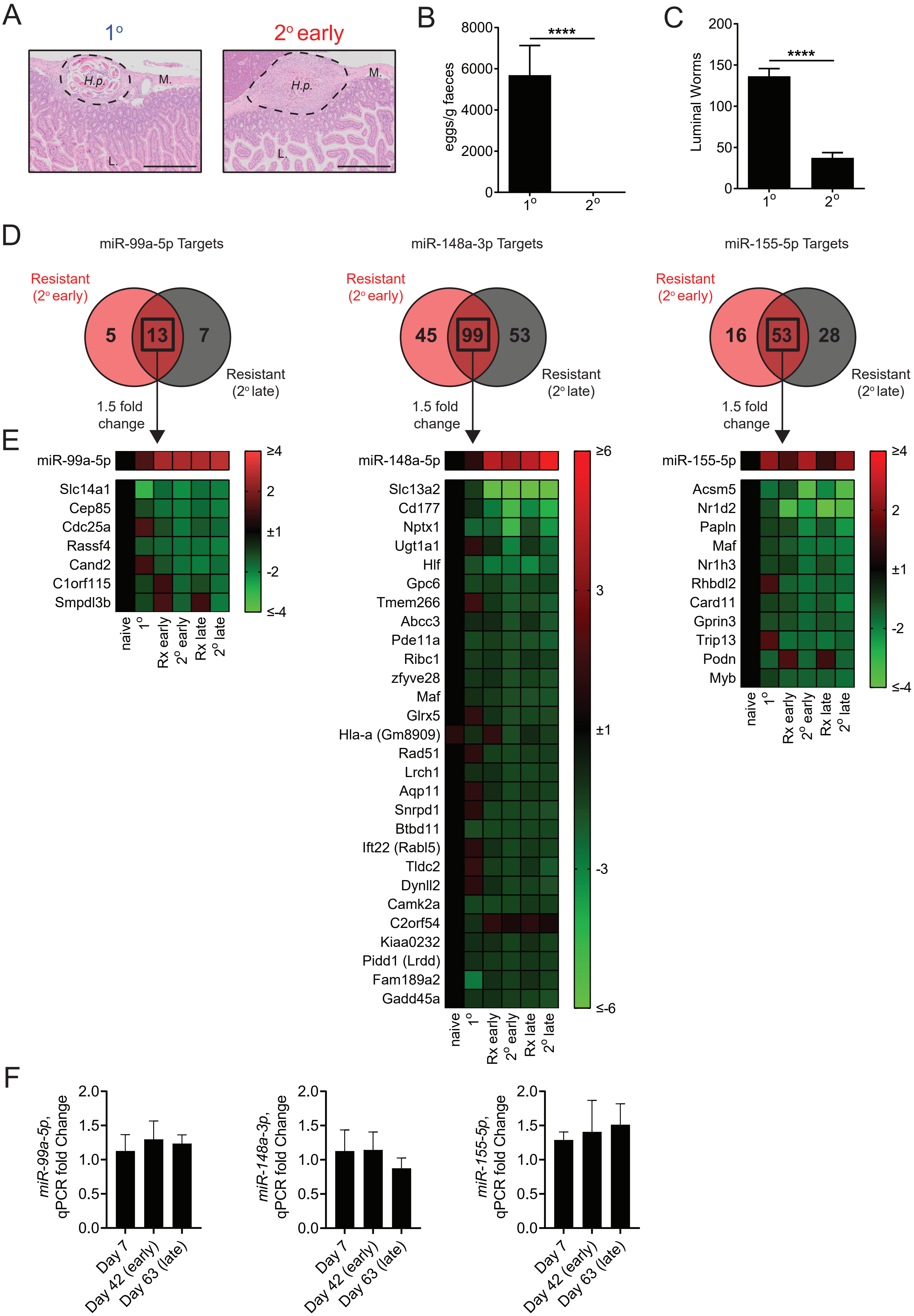

Figure S2: Single miRNA inhibition does not perturb key antihelminth immune and effector responses

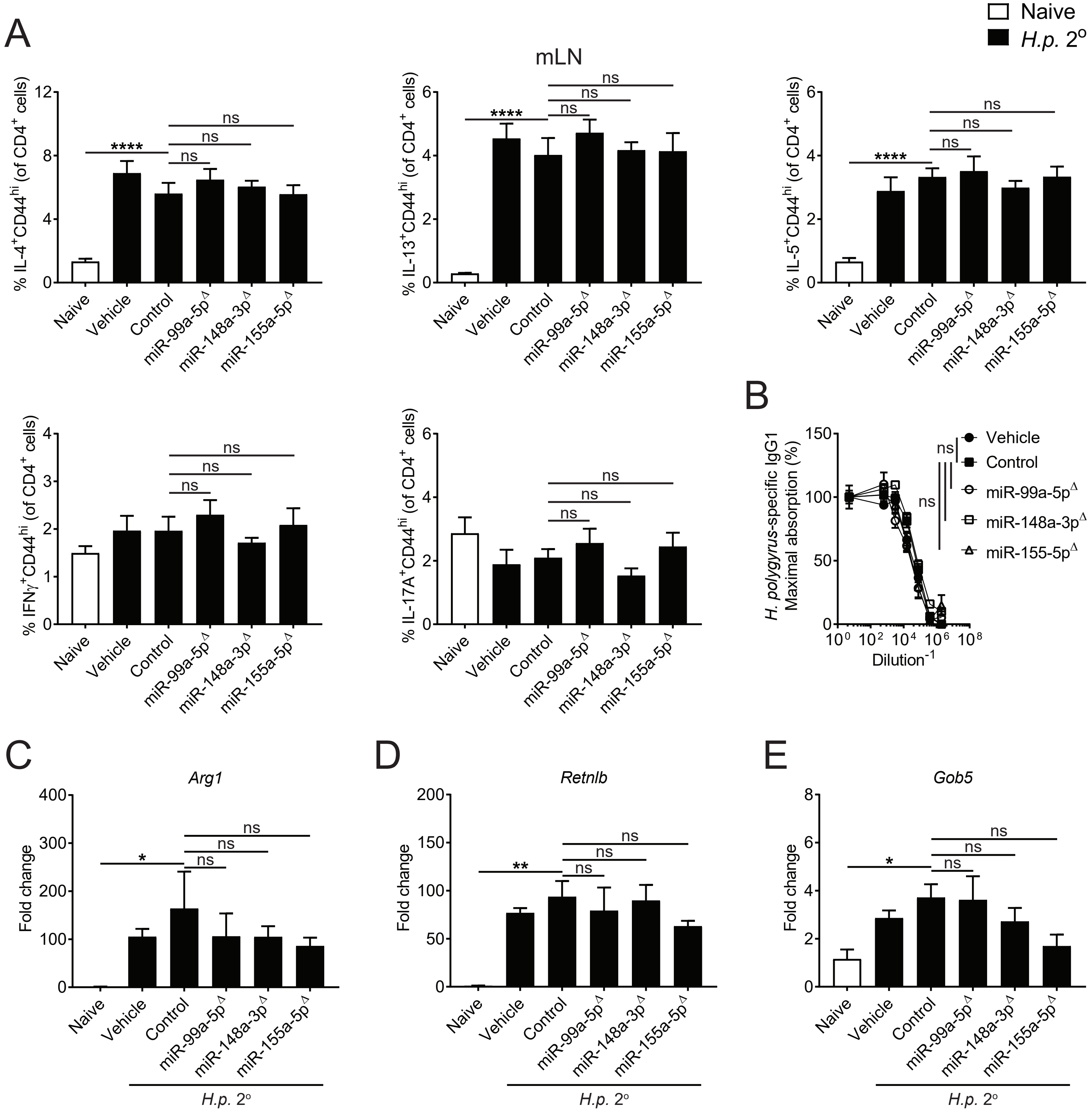

Figure S3: Triple miRNA inhibition does not perturb key antihelminth immune and reffector responses

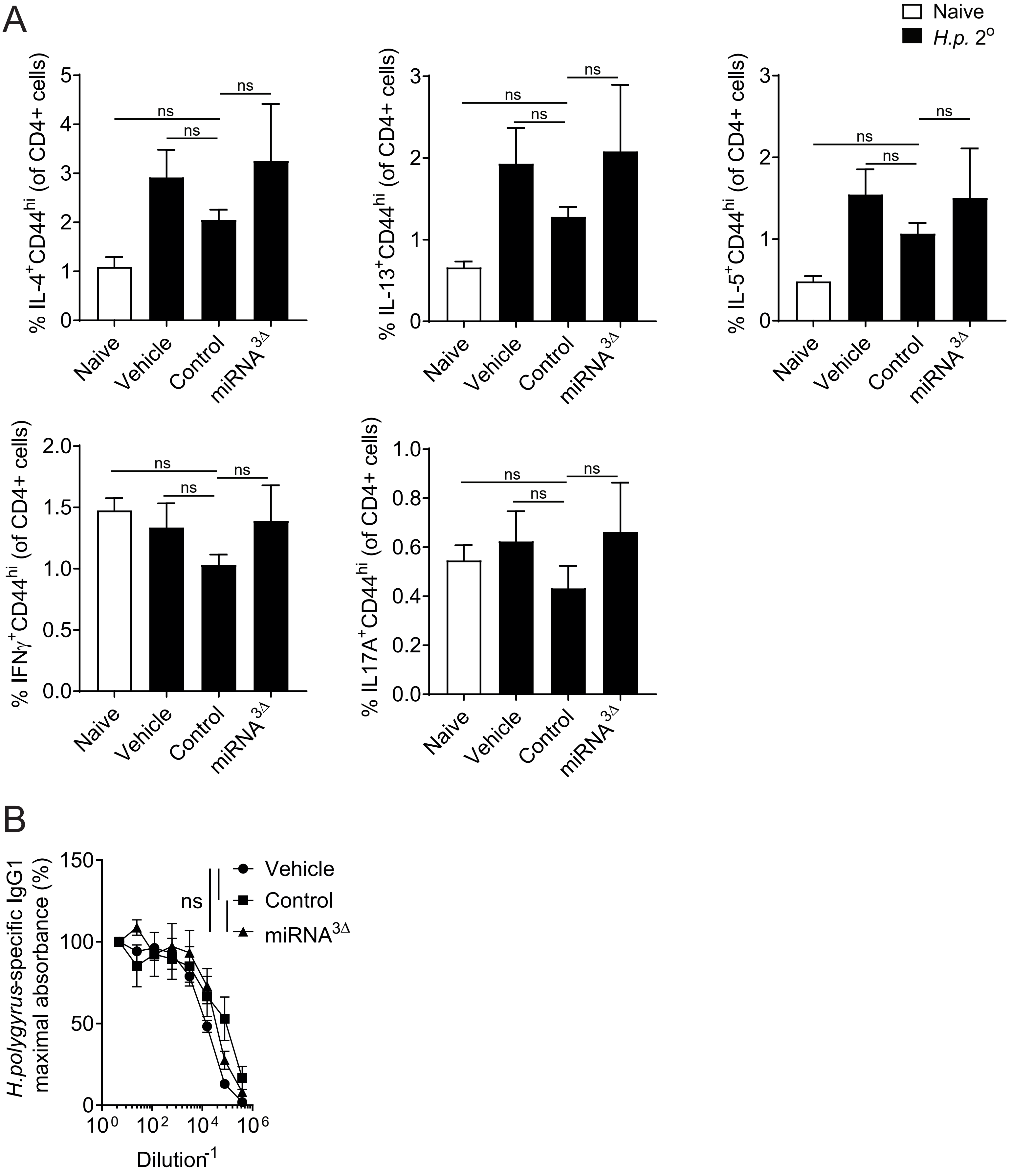

Figure S4: miR-99a-5p and miR-155-5p miRNA inhibition abrogates allergic airway inflammation

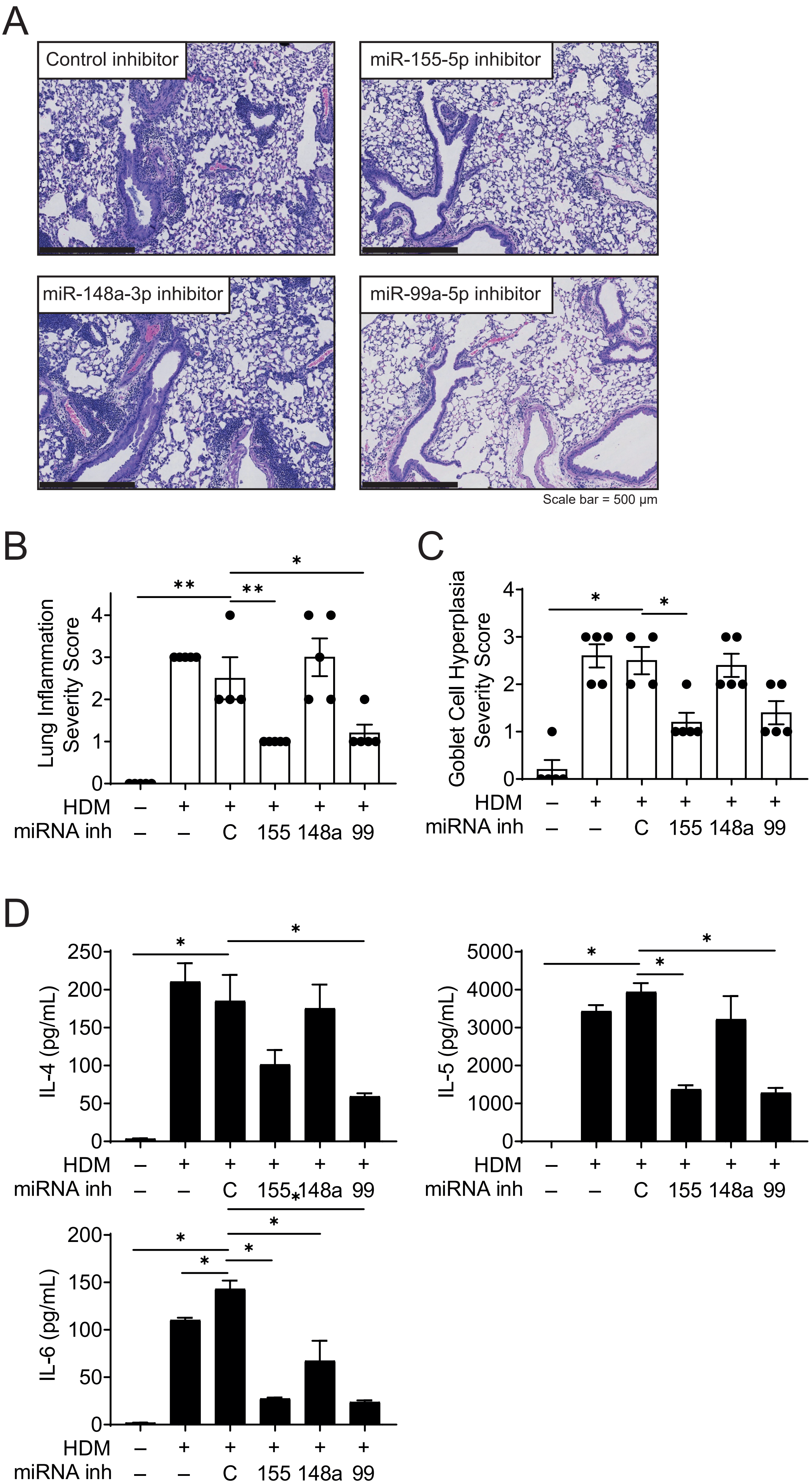

Figure S5: Pathway analysis of lung transcriptome following miRNA inhibition

A

Category	Canonical Pathways	HDM+control Inhibitor	HDM+miR-155 inhibitor	HDM+miR-148a inhibitor	HDM+miR-99a inhibitor
Adaptive Immunity	Role of NFAT in Regulation of the Immune Response	5	3	5.477	2
	PKCθ Signaling in T Lymphocytes	4.69	2.828	4.811	N/A
	PKCθ Signaling in T Lymphocytes	4.69	2.828	4.811	N/A
	iCOS-iCOSL Signaling in T Helper Cells	3.873	2.236	4.243	N/A
	iCOS-iCOSL Signaling in T Helper Cells	3.873	2.236	4.243	N/A
	Systemic Lupus Erythematosus In T Cell Signaling Pathway	3.411	N/A	3.402	N/A
	CD28 Signaling in T Helper Cells	3.317	2	3.606	N/A
	Th1 Pathway	3.273	2.828	3.78	N/A
	Systemic Lupus Erythematosus In B Cell Signaling Pathway	3	N/A	2.967	N/A
	Th2 Pathway	2.84	1.897	2.985	N/A
	Calcium-induced T Lymphocyte Apoptosis	2.673	2.449	2.668	N/A
	Th17 Activation Pathway	2.111	N/A	2.309	N/A
	Cytotoxic T Lymphocyte-mediated Apoptosis of Target Cells	2	2	2.236	N/A
Innate Immunity	Dendritic Cell Maturation	4.49	N/A	5.284	N/A
	Fcγ Receptor-mediated Phagocytosis in Macrophages and Monocytes	3.207	N/A	2.837	N/A
	Role of Pattern Recognition Receptors in Recognition of Bacteria and Viru	2.84	2	2.668	N/A
	MIF Regulation of Innate Immunity	2.828	N/A	2.646	N/A
	iNOS Signaling	2.449	N/A	2.449	N/A
	Crosstalk between Dendritic Cells and Natural Killer Cells	2.333	N/A	3.051	N/A
	Fc Epsilon RI Signaling	2.121	N/A	1.941	N/A
	Production of Nitric Oxide and Reactive Oxygen Species in Macrophages	2.065	N/A	2.558	N/A
Signaling pathways	TREM1 Signaling	3.153	N/A	3.578	N/A
	Tec Kinase Signaling	2.84	N/A	2.982	N/A
	NF-κB Activation by Viruses	2.53	N/A	2.111	N/A
	Role of IL-17F in Allergic Inflammatory Airway Diseases	2.236	N/A	2.236	N/A
	IL-8 Signaling	2.183	N/A	2.294	N/A
	SAPK/JNK Signaling	2	N/A	2.333	N/A
	LPS/IL-1 Function	2	N/A	2.236	N/A
	GM-CSF Signaling	2	N/A	2.236	N/A
	TNFR1 Signaling	2	N/A	0.816	N/A
Neuroinflammation	Neuroinflammation Signaling Pathway	2.828	N/A	3.363	N/A
Cell migration/recruitment	Integrin Signaling	2.496	N/A	2.524	N/A
	Leukocyte Extravasation Signaling	2.4	N/A	3.157	N/A
	Chemokine Signaling	2	N/A	2	N/A
	Paxillin Signaling	2	N/A	1.633	N/A
Metabolic pathways	Unfolded protein response	2.449	N/A	2.449	N/A
	Citrulline Metabolism	2	N/A	2	N/A
	Prostanoid Biosynthesis	2	N/A	N/A	N/A
